# CRISPR-CISH: an in situ chromogenic DNA repeat detection system for research and life science education

**DOI:** 10.1007/s10577-025-09767-1

**Published:** 2025-04-22

**Authors:** Bhanu Prakash Potlapalli, Fabian Dassau, Jörg Fuchs, Deboprio Roy Sushmoy, Andreas Houben

**Affiliations:** 1 Leibniz Institute of Plant Genetics and Crop Plant Research (IPK) Gatersleben, Corrensstraße 3, 06466 Seeland, Germany; 2 Hochschule Mittweida, Department of Biotechnology, 09648 Mittweida, Germany; 3 Julius Kühn-Institute (JKI), 06484 Quedlinburg, Germany

**Keywords:** CRISPR-CISH, Cas9, Chromsome, CRISPR-FISH, Chromogenic detection

## Abstract

In situ hybridization is a technique to visualize specific DNA sequences within nuclei and chromosomes. Various DNA in situ fluorescent labeling methods have been developed, which typically involve global DNA denaturation prior to the probe hybridization and often require fluorescence microscopes for visualization. Here, we report the development of a CRISPR/dCas9-mediated chromogenic in situ DNA detection (CRISPR-CISH) method that combines chromogenic signal detection with CRISPR imaging. This non-fluorescent approach uses 3' biotin-labeled tracrRNA and target-specific crRNA to form mature gRNA, which activates dCas9 to bind to target sequences. The subsequent application of streptavidin alkaline phosphatase or horseradish peroxidase generates chromogenic, target-specific signals that can be analyzed using conventional bright-field microscopes. Additionally, chromatin counterstains were identified to aid in the interpretation of CRISPR-CISH-generated target signals. This advancement makes in situ DNA detection techniques more accessible to researchers, diagnostic applications, and educational institutions in resource-limited settings.

**Supplementary Information:**

The online version contains supplementary material available at 10.1007/s10577-025-09767-1.

## Introduction

**Fig. 1:**
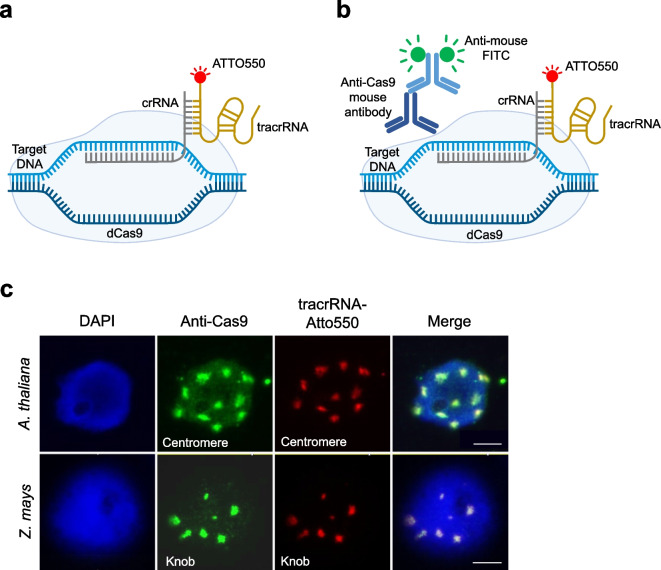
Indirect labeling of DNA repeats by combining CRISPR-FISH with indirect immunostaining. **a** Schematic of standard CRISPR-FISH method. **b** Schematic of indirect CRISPR-FISH to label repeat sequences with Cas9 antibody and later detection with anti-mouse FITC. **c** Visualization of centromere repeats in *A. thaliana* (upper) and knob repeats in *Z. mays* (lower) using CRISPR-FISH and immunofluorescence. Cas9 antibody was detected with anti-mouse FITC (green) and tracrRNA was labeled with Atto550 (red). DNA stained with DAPI (blue). Scale bars, 5 μm

Fluorescence in situ hybridization (FISH) is a versatile tool for localizing specific nucleic acid sequences in fixed cells and tissues using a fluorescence microscope. Chromogenic in situ hybridization (CISH) is an alternative method that detects sequences through chromogenic signal detection, making it compatible with conventional bright-field microscopes (Brigati et al. 1983). The major drawback of standard in situ hybridization methods is the need for high temperatures or formamide treatments to denature DNA for probe hybridization, which can damage the chromatin structure (Kozubek et al. 2000; Shim et al. 2024). Moreover, the hybridization and subsequent wash steps in standard in situ hybridization protocols extend the time required to obtain results. To address these issues, a straightforward CRISPR/Cas9-based method called CRISPR-FISH (synonym REGEN-ISL, Ishii et al. 2019) was developed for labeling DNA repeats in fixed plant and animal specimens (Ishii et al. 2019; Potlapalli et al. 2020). CRISPR-FISH uses a target-specific crRNA and a universal tracrRNA with a single fluorescent dye added to the 5'end and dCas9 to label the target sequence (Fig. 1a).

Unlike most in situ hybridization methods, CRISPR-FISH does not require a global DNA denaturation, ensuring a better preservation of the chromatin structure (Němečková et al. 2019). This makes it an excellent tool for studying the three-dimensional genome organization with super-resolution imaging (Deng et al. 2015; Ishii et al. 2019; Němečková et al. 2019; Potlapalli et al. 2020). The application of the dCas9-RNA complex enables rapid detection of repetitive DNA sequences within seconds, highlighting the fast labeling efficiency of CRISPR-FISH (Ishii et al. 2019). CRISPR-FISH allows multicoloured labeling of repeats in various fixation conditions and specimen samples and works in a large temperature range (4—37 ºC) (Ishii et al. 2019; Potlapalli et al. 2020; Nagaki and Yamaji 2020). Recently, the labeling intensity of CRISPR-FISH has been improved by integrating the ALFA-tag and TSA approaches, making it an even more versatile imaging tool (Potlapalli et al. 2024). However, broader implementation of this method faces challenges in resource-limited laboratories that lack access to fluorescence microscopes. To overcome this limitation, we developed a robust non-fluorescent CRISPR-labeling method for detecting high-copy repeats. The newly developed **CRISPR**/Cas9 mediated **c**hromogenic **i**n **s**itu **h**ybridization method (CRISPR-CISH) combines chromogenic signal detection methods using alkaline phosphatase or horseradish peroxidase with CRISPR-imaging. The distribution of chromogenic signals can be analyzed by conventional bright-field microscopes. High-copy sequences of mouse and several plant species (*Arabidopsis thaliana, Zea mays*, *Allium fistulosum, Allium cepa* and *Vicia faba*) were used to demonstrate the effectiveness of the developed in situ DNA detection method. To assist educators in conducting CRISPR-CISH in their classrooms, a simplified graphical hands-on protocol for schools is provided here.

## Materials and methods

Nuclei and chromosomes from *Arabidopsis thaliana*, maize (*Zea mays*), Welsh onion (*Allium fistulosum*)*,* onion (*Allium cepa*), broad bean (*Vicia faba*), and the house mouse (*Mus musculus forma domestica*) were used.

### Preparation of leaf nuclei

Young leaf tissue was fixed in formaldehyde (2% or 4%) in ice-cold Tris buffer (10 mM Tris–HCl, 10 mM Na_2_-EDTA, 100 mM NaCl, 0.1% Triton X- 100, and adjusted to pH 7.5 with NaOH) under vacuum for 5 min and followed by incubation for 25 min on ice without vacuum. The tissue was rinsed twice in ice-cold Tris buffer for 5 min each. Then, the tissue was chopped in a Petri dish in 300—500 µl ice-cold LB01 buffer (15 mM Tris–HCl (pH 7.5), 2 mM Na_2_-EDTA, 0.5 mM spermin, 80 mM KCl, 20 mM NaCl, 15 mM β-mercaptoethanol and 0.1% Triton X- 100) using a fresh razor blade. The suspension was filtered through a 35 µm cell-strainer cap (BD Biosciences) and spun onto glass slides using a Cytospin 3 (Shandon) with 700 rpm for 5 min. The slides were either used immediately or kept in 1 × PBS at 4 °C overnight. Alternatively, slide preparation with formaldehyde-fixed cells could be performed using a sucrose buffer instead of a cytospin, as described by (Potlapalli et al. 2023).

### Preparation of chromosomes

Root tip meristems were obtained from 3—5 days old *Z. mays, V. faba* and *A. cepa* seedlings and potted *A. fistulosum* plants. *Z. mays and V. faba* roots were treated in 0.1% colchicine at room temperature (RT) for 3 h. *A. cepa* and *A. fistulosum* roots were treated in ice-cold water for 24 h. Then, roots of all species were fixed in 3:1 (ethanol: acetic acid) solution for 24 h at RT and washed twice in ice-cold ddH_2_0 and 0.01 M citrate buffer (0.01 M sodium citrate and 0.01 M citric acid, pH 4.5–4.8), for 5 min each. 3—5 root tips of *Z*. *mays, V. faba, and A. fistulosum* were digested for 50, 60 and 60 min, respectively in 25–50 µl of enzyme mixture (0.7% cellulase R10, 0.7% cellulase, 1% pectolyase, 1% cytohelicase dissolved in 0.01 M citrate buffer) depending on the number of root tips at 37 ⁰C. The tubes with digested meristems were vortexed and, after adding 600 µl of ddH_2_O centrifuged at 10,000 rpm for 45 s. The supernatant was removed, and 600 µl 96% ethanol was added and centrifuged at 11,000 rpm for 30 s. After the supernatant was discarded by inverting the tubes, the pellets were resuspended in 80–100 µl 96% ethanol. For the preparation of chromosome slides, 10 µl of cell suspension was dropped from a height of 10–15 cm onto the glass slide in a moisture box placed on a hot plate at 55° C. Then 25–50 µl of freshly prepared ethanol: acetic acid (3:1) at RT was added to the glass slide and air dried.

*A. cepa* chromosomes were prepared using the squash technique. A 3:1-fixed single root was incubated in 45% acetocarmine in acetic acid for 5 min. The root cap was removed, and the meristematic tip was squeezed onto a slide. After adding 10—15 µl of 45% acetic acid, the sample was covered with a coverslip and gently tapped. Brief heating was applied with a spirit burner from beneath the slide, followed by freezing with liquid nitrogen. The coverslip was then removed, and the slide was immediately immersed in 99% ethanol and then air-dried.

*A. thaliana* chromosomes were obtained from young flower buds. The flower buds were fixed in 3:1 (ethanol: acetic acid) solution for 24 h at RT. Then, the flower buds were washed twice in ddH_2_0 and buds containing yellow anthers were removed. The remaining buds were washed twice in 0.01 M citrate buffer for 5 min each on ice and digested with a 50% enzyme mixture (0.35% cellulase R10, 0.35% cellulase, 0.5% pectolyase, 0.5% cytohelicase dissolved in 0.01 M citrate buffer) in 1 × citrate buffer at 37 ⁰C for 75 min. Later, the enzyme mixture was replaced with 0.01 M citrate buffer. For the preparation of chromosome slides, a single flower bud was placed onto a glass slide and 10—20 µl of 60% acetic acid was added. The slide was placed on a hot plate (50 ⁰C) and the suspension was spread by circular stirring with a dissecting needle for 30 s (Mandáková and Lysak 2016). Later, 25—50 µl of freshly prepared ethanol: acetic acid (3:1) was added to the glass slide and dried. Slides containing well-spread chromosomes were selected using a phase-contrast light microscope.

Murine chromosomes were obtained from the skin of a laboratory house mouse strain C57Bl6/J. Chromosome slide preparation was done as described in (Potlapalli et al. 2020).

### Preparation of recombinant dCas9

The *Streptococcus pyogenes* (dead version) Cas9 gene sequence containing the double nuclease mutation (D10 A and H840 A) was PCR-amplified from the dCas9:3 × PP7:GFP vector (Khosravi et al. 2020) and cloned into pET22b + (Invitrogen) with a C-terminal hexahistidine affinity tag. The plasmid was transformed into *Escherichia coli* BL21 (DE3) and grown in 2 × TY media at 37 °C to OD600 ~ 0.5 and then induced by 0.5 mM isopropyl-β-D- 1-thiogalactopyranoside (IPTG) for 16 h at 18 °C. Cells were lysed in lysis buffer (50 mM NaH_2_PO_4_, 500 mM NaCl, 10% glycerol, 10 mM imidazole, pH 8) containing lysozyme (1 mg/ml) for 30 min on ice, then snap frozen in liquid nitrogen and immediately thawed in water at RT followed by sonication on ice for 4 times for 30 s each at 50% intensity using a Vibra-Cell sonicater Model VC60 (Sonics & Materials Inc.). The lysate was centrifuged at 7000 rpm for 20 min at 4 °C, and the supernatant was transferred to a Falcon tube containing 1 ml of PureCube 100 Ni–NTA Agarose (Cube Biotech, 31103) and rotated at 4 °C for 90 min. The His-tagged dCas9 protein was then purified by gravity flow chromatography by passing the supernatant through disposable polypropylene columns (Qiagen, 34924) and eluted with elution buffer (50 mM NaH_2_PO_4_, 500 mM NaCl, 10% glycerol, 250 mM imidazole, pH 8). Purified dCas9 protein was stored at -20 °C. Alternatively, recombinant dCas9 can be commercially purchased, e.g. from Novateinbio (Catalog: PR-137213).

### Standard CRISPR-FISH

We employed the bipartite gRNA (crRNA and 3'ATTO550/Alexa Fluor488-labeled tracrRNA) system (Alt-R® CRISPR-Cas9, Integrated DNA Technologies, https://eu.idtdna.com) for CRISPR-FISH (synonym REGEN-ISL, Ishii et al. 2019). Target-specific crRNA were designed using the software CRISPRdirect (https://crispr.dbcls.jp/) (Supplementary Table). Preparation of the RNP complex was done using the standard protocol (Ishii et al. 2019). Prepared slides were washed with 0.2% Triton-X100 in Tris–HCL (pH 9) at 37 °C for 1–2 h, and later washed in 1 × PBS twice for 5 min each. 100 µl of 1 × Cas9/1 mM DTT buffer (200 mM Hepes (pH 7.5), 1 M KCl, 50 mM MgCl_2_, 50% glycerol, 10% BSA, and 1% Tween 20) was added per slide for 5 min at RT and then buffer was removed by tilting the slides. 20—25 µl of RNP complex was added per slide and covered with parafilm and incubated at 37 ºC for 1 h in a moisture box. After incubation, the slides were washed in 1 × PBS for 5 min and post-fixed with 4% formaldehyde in 1 × PBS for 5 min at RT. Then the slides were washed with 1 × PBS and dehydrated in an ethanol series (70%, 90%, and 96%) for 2 min each. Finally, slides were air-dried in the dark, and counterstained with 8 µl of (0.5 μg/ml) 4′,6-diamidino-2-phenylindole (DAPI) in antifade (Vector Laboratories, Burlingame, CA, USA) and stored at 4 ºC for further microscopy.

### Indirect CRISPR-FISH employing an anti-Cas9 antibody

After standard CRISPR-FISH, slides were blocked with 100 µl of 4% BSA for 1 h at RT in the dark. Then 50 µl of a monoclonal anti-Cas9 mouse antibody (Santa Cruz Biotechnology, 7 A9 - 3 A3) diluted 1:300 in 2% BSA in 1 × PBS was added per slide and incubated at 37 ºC for 1 h and subsequently overnight at 4 ºC in a humid box. The slides were washed in 1 × PBS twice for 5 min on ice. Then 50 µl of secondary anti-mouse Alexa 488 antibody (Thermo Fisher Scientific Inc. Massachusetts, U.S.A. cat: A11001) diluted 1:200 in 2% BSA in 1 × PBS was applied per slide and incubated at 37 ºC for 1 h. Subsequently, the slides were washed twice with ice-cold 1 × PBS for 5 min. Dehydration and counterstaining were performed as described for standard CRISPR-FISH.

### Indirect CRISPR-FISH using biotinylated tracrRNA

A 3'biotinylated tracrRNA (Integrated DNA Technologies, https://eu.idtdna.com, Supplementary Table) and target-specific crRNA were used for the preparation of the RNP complex using a standard protocol (Ishii et al. 2019). CRISPR-FISH was performed as described for standard CRISPR-FISH until post-fixation and washing for 5 min in 1 × PBS at RT. Afterwards, the slides were blocked with 100 µl of 4% BSA in 1 × PBS for 30 min at RT and then washed twice in 1 × PBS for 5 min each. Then 50 µl of streptavidin-conjugated FITC (Sigma-Aldrich, S3762) diluted 1:40 in 2% BSA/1 × PBS was added per slide and incubated at 37 ºC for 1 h. Subsequently, the slides were washed twice in 1 × PBS for 5 min each at RT in dark and dehydrated and counterstained as described for standard CRISPR-FISH.

### CRISPR-CISH—CRISPR Cas9 mediated chromogenic in situ detection

CRISPR-CISH was performed like indirect CRISPR-FISH using biotinylated tracrRNA till post-fixation and washing for 5 min in 1 × PBS at RT. The slides were blocked with 100 µl of 4% BSA in 1 × PBS for 30 min at RT and then washed twice in 1 × PBS for 5 min each. Then 50 µl of streptavidin-conjugated alkaline phosphatase (AP) (ZytoChem Plus AP Kit, AP008RED) or horseradish peroxidases (HRP) (Permanent HRP Green Kit, ZUC070 - 100) per slide was added according to manufacturer’s instructions and incubated at 37 ºC for 1 h. Then, the slides were washed twice in 1 × PBS for 5 min each at RT. In the case of AP, 50 µl of permanent red buffer with 0.8 µl permanent red concentrate (ZytoChem Plus AP Kit, AP008RED) was added and incubated at RT until the red color developed (usually around 10 min, the precise time should be figured out empirically). In the case of HRP, 50 µl of HRP green substrate buffer with 4.5 µl HRP green chromogen (Permanent HRP Green Kit, ZUC070 - 100) was added and incubated at RT until the green color developed (over 10 min). The desired color intensity was monitored using a light microscope. Then, the slides were washed in ddH_2_O at sufficient color intensity. The slides were counterstained with a nuclear dye and later rinsed with tap water and air-dried. Finally, slides were mounted with Entellan® (Merck KGaA) and analyzed by light microscopy.

### Standard fluorescence in situ hybridization (FISH)

FISH with a 5`biotin-labeled *A. thaliana* centromere-specific (Murata et al. 1994) and telomere-specific oligo probes (Dreissig et al. 2017) (Supplementary Table) was performed as described in (Ma et al. 2008). Nuclei-carrying slides were prepared as mentioned for CRISPR-FISH. Then, they were washed with 2 × SSC for 5 min each and further treated with 45% acetic acid for 10 min at RT. Afterwards, slides were washed in 2 × SSC for 5 min each and fixed in 4% formaldehyde in 1 × PBS for 10 min at RT. To remove the excess of fixative, the slides were washed thrice with 2 × SSC for 4 min each and dehydrated in an ethanol series (70%, 90%, and 96%) for 2 min each, followed by air drying. 20 μl of hybridized buffer (50% deionized formamide, 20 × SSC, 10% dextran) containing probe was added on each slide under a 22 × 22 coverslip and denatured at 80 ºC for 2 min on a hot plate and incubated overnight at 37 ºC in a moisture box. On the next day, the slides were washed twice in 2 × SSC for 5 min each to remove the coverslip. To remove the unspecifically bound probes, slides were washed with 2 × SSC at 58 ºC for 20 min in a water bath and later transferred to 2 × SSC at RT for 2 min. Then the slides were incubated with 100 μl of blocking solution (4% BSA, 0.1% Tween 100, 1 × PBS) under parafilm for 30 at RT and washed twice for 5 min each in 2 × SSC at RT. Afterwards, slides were probed with streptavidin-conjugated to FITC (Sigma-Aldrich) (1:100 diluted) for 1 h at 37 °C under parafilm in a humid chamber, followed by washing thrice for 5 min each in 2 × SSC at RT in the dark and dehydrating in an ethanol series (70%, 90%, and 96%) for 2 min each. Finally, the slides were air-dried in the dark and counterstained with 8 µl of (0.5 μg/ml) 4′,6-diamidino- 2-phenylindole (DAPI) in antifade (Vector Laboratories, Burlingame, CA, USA) and stored at 4 ºC for further microscopy.

### Counterstaining of specimens with nuclear dyes

Methylene green, methyl green (Sigma, M8884 - 5G), neutral red (Feinchemie K.-H. Kallies KG), and naphthol green B (Waldeck, 1B- 385) solutions were prepared in ddH_2_O. In addition, 100% stock solutions of hematoxylin (Sigma, GHS3 - 50ML), and methylene blue (Merck, 115,943) were tested. 300 µl of dye was added to each slide for counterstaining.

### Microscopy

Chromogenic in situ imaging was performed using an Axiophot light microscope (Carl Zeiss) equipped with an Axiocam 506 color camera and operated using Zen 3.0 blue Edition software (Carl Zeiss). Fluorescence imaging was performed using a BX61 microscope (Olympus) equipped with an ORCA-R2 CCD camera (Hamamatsu) and operated using cellSens imaging software. All images were captured in grayscale and later pseudo-colored using ImageJ software (https://imagej.nih.gov/ij/).

### Signal quantification

For quantification of signals, leaf tissues of *A. thaliana* and *Z. mays* were fixed in 4% and 2% formaldehyde in TRIS buffer, respectively (Ishii et al. 2019; Němečková et al. 2019), respectively. Nuclei were isolated and 2 C nuclei were sorted using a BD Influx cell sorter (BD Biosciences) according to (Ahmadli et al. 2022). CRISPR-imaging signals from 50 flow-sorted 2 C nuclei per each analysis were counted.

## Results

### Application of anti-Cas9 for the detection of dCas9-binding sites

High-copy DNA repeats in fixed chromosomes and nuclei can be fluorescently labeled using CRISPR-FISH (Ishii et al. 2019; Potlapalli et al. 2020). To explore the possibility of using this CRISPR-based approach to detect repeats non-fluorescently; first, a new CRISPR-FISH strategy had to be developed to label DNA indirectly, as non-fluorescent labeling of DNA can only be achieved indirectly. Therefore, we aimed to test first an indirect CRISPR-FISH method by combining immunofluorescence with standard CRISPR-FISH. In this approach, ATTO550-tagged bipartite gRNA-guided CRISPR-FISH was combined with a Cas9-specific antibody and an anti-mouse conjugated FITC to indirectly visualize dCas9-binding sites (Fig. 1b). Using this method, *A. thaliana* centromere and *Z. mays* knob repeats were detected using previously reported crRNAs (Fig. 1c) (Ishii et al. 2019; Němečková et al. 2019). The overlap of indirect anti-Cas9 (green) and tracrRNA-Atto (red) signals proved the functionality of the tested indirect CRISPR-FISH method (Fig. 1c).

### Replacing fluorescence- with non-fluorescence-based labeling of dCas9-binding sites

**Fig. 2:**
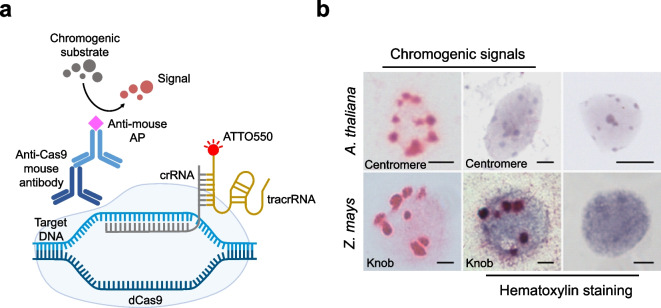
Non-fluorescent labeling of DNA repeats by combining CRISPR-FISH and indirect immunostaining. **a** Schematic of CRISPR-FISH for non-fluorescent labeling of repeat sequences using Cas9 antibody and anti-mouse alkaline phosphatase (AP) and application of red chromogenic substrate develops red colored precipitate at RNP bound sites. (**b**, upper) non-fluorescent labeling of centromere repeats in *A. thaliana* nuclei (left) without staining, (middle) with hematoxylin counterstaining and (right) with counterstaining only as control without labeling. In *A. thaliana* nuclei, hematoxylin also stained the chromocenters where the actual centromeres were located, so the red chromogenic centromeric dots were masked by hematoxylin. (**b**, lower) non-fluorescent labeling of knob repeats in *Z. mays* nuclei (left) without staining, (middle) with hematoxylin counterstaining and (right) with counterstaining only as control without labeling. Red-colored spots indicate centromere and knob-specific signals. Scale bars, 5 μm

To replace the fluorescence-based detection of anti-Cas9 binding sites with a non-fluorescence method, we employed alkaline phosphatase (AP) for the detection of the target sequences (Fig. 2a). In this approach, anti-mouse conjugated alkaline phosphatase was used to detect anti-Cas9 binding sites. The application of a red chromogenic substrate reacted with alkaline phosphatase, resulting in the expected numbers of red-colored, non-fluorescent signals of centromeres and knob repeat clusters in formaldehyde-fixed nuclei of *A. thaliana* and *Z. mays*, respectively (Fig. 2b). Hematoxylin was used to counter stain chromatin.

However, this dye resulted even in the absence of CRISPR-dCas9 labeling in pronounced staining of the centromeric chromocenters in *A. thaliana* (Fig. 2b, upper right), obscuring the detection of the CRIPSR-based centromere signals (Fig. 2b, upper middle). Therefore, haematoxylin is not a suitable counterstain for *A. thaliana* nuclei when combined with a non-fluorescent CRISPR-ISH method. In contrast, *Z. mays* nuclei without CRISPR-dCas9 labeling resulted in a more uniform haematoxylin staining (Fig. 2b, lower right). The same chromogenic approach without subsequent counterstaining was used to detect *Fok*I and sub-telomeric repeats in *V. faba* and *A. fistulosum* nuclei, respectively (Supplementary Fig. 1a). The observed distinct signal clusters of different sizes were similar to those obtained by CRISPR-FISH control experiments (Supplementary Fig. 1b) with the difference that the non-fluorescent CRISPR-ISH generated a higher background noise level. However, microscopic evaluation of several slides revealed that in all cases, independent of the analysed species and sequence, only a few nuclei (up to 7.5%) showed the target-specific signals. This indicates the low efficiency of the indirect detection method using the Cas9 antibody in combination with anti-mouse conjugated alkaline phosphatase. Additionally, these results underscored the need to investigate appropriate nuclear stains across different species to complement non-fluorescent CRISPR-ISH labeling.

### Identification of suitable chromatin counterstains

To enable a correct interpretation of the CRISPR-ISH signals within the analysed nuclei or chromosomes, we tested chromatin dyes suitable for conventional bright-field microscopy. Out of the six tested dyes, 4% methylene green, 2% methyl green, 4% neutral red, hematoxylin (100% stock solution) and methylene blue (100% stock solution) successfully stained formaldehyde fixed nuclei of *Z. mays*, *V. faba* and *A. fistulosum* within 2 min at RT (Supplementary Fig. 2a). Among these, only neutral red and hematoxylin provided clear staining of the nuclear periphery, enabling consistent identification of nuclei across all three species. These two dyes were selected for further experiments. In *A. thaliana,* however, only hematoxylin (100% stock solution) and methylene blue (100% stock solution) effectively stained the nuclei within 10 min of dye application (Supplementary Fig. 2b). Hematoxylin provided stronger staining compared to methylene blue, but both dyes intensely stained chromocenters (Supplementary Fig. 2b), rendering them unsuitable as they could obscure CRISPR-ISH signals. Thus, we decided not to use a chromatin dye in *A. thaliana* for the subsequent experiments. In general, to achieve a clear contrast between CRISPR-ISH signals and the chromatin stain, it is essential to select an appropriate counterstain based on the chromogenic substrate used. Moreover, our observations revealed a likely correlation between the counterstaining ability of chromatin and the genome size of the species. For instance, the small-genome species *A. thaliana* (142.5 Mb/1 C; https://www.ncbi.nlm.nih.gov/datasets/genome/GCA_028009825.2/) exhibited weaker chromatin staining compared to the large-genome species *V. faba* (13 Gb/1 C) (Jayakodi et al. 2023) and *A. fistulosum* (11.27 Gb/1 C) (Liao et al. 2022).

### The application of biotinylated tracrRNA improved indirect CRISPR-ISH and enabled CRISPR-CISH

**Fig. 3:**
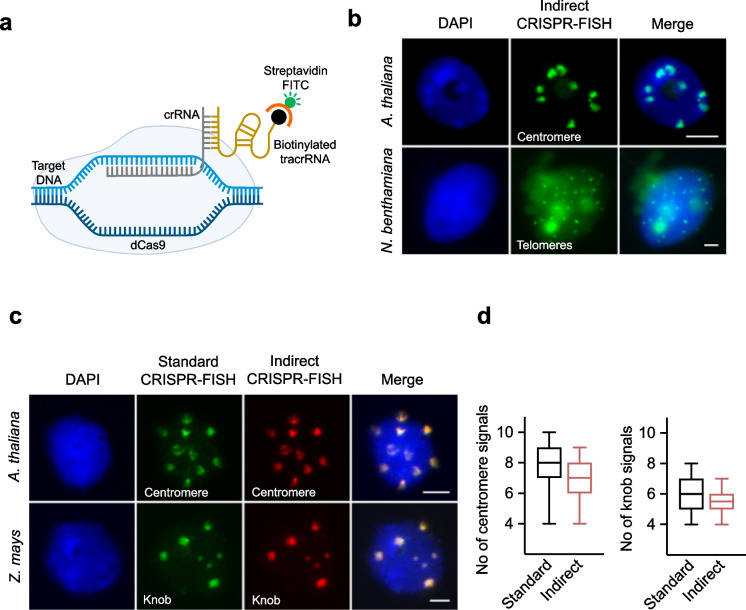
Indirect CRISPR-FISH labeling of DNA repeats using biotinylated tracrRNA. **a** Schematic of indirect CRISPR-FISH for the labeling of repeat sequences using biotinylated tracrRNA and detection with streptavidin FITC. **b** Visualization of (upper) centromere repeats in *A. thaliana* and (lower) telomere repeats in *N. benthamiana* (bottom) with indirect CRISPR-FISH using biotinylated tracrRNA and detected with streptavidin FITC. **c** Images showing colocalization of indirect CRISPR-FISH (green) and standard CRISPR-FISH (red) spots labeling centromeres and knob repeats in *A. thaliana* and *Z. mays* nuclei, respectively. Biotinylated tracrRNA was detected using streptavidin FITC (green). DNA stained with DAPI (blue). Scale bars, 5 µm. **d** Comparison of labeling efficiency between two methods, number of (left) centromere and (right) knob signals generated per nucleus by standard CRISPR-FISH and indirect CRISPR-FISH methods, *n* = 50 nuclei. Upper and lower whiskers indicate the maximum and minimum number of signals observed. The black line in the middle of each box plot indicates the median

To increase the efficiency of the indirect CRISPR-ISH approach, we first employed 3'biotin-labeled tracrRNA in combination with streptavidin-conjugated FITC (Fig. 3a). To assay the functionality of this approach, *Arabidopsis* centromere- and *Arabidopsis*-type telomere-specific crRNAs were applied and formaldehyde-fixed *A. thaliana* and *N. benthamiana* nuclei showed target sequence-specific labeling, respectively (Fig. 3b). As a positive control, standard CRISPR-FISH using Atto550-labeled tracrRNA and standard FISH with biotin-labeled oligo-probes specific for both sequences were used (Supplementary Fig. 3a & b). In addition, the specificity of the indirect CRISPR-FISH signals was demonstrated by probing *A. thaliana* centromere and *Z. mays* knob repeats by simultaneous application of the standard CRISPR-FISH and indirect CRISPR-FISH (Fig. 3c)*.* To compare the labeling efficiency of both methods, centromere and knob signals of 2 C sorted nuclei generated by both standard and indirect CRISPR-FISH were quantified. In the counting assay, about 8 and 7 centromere-specific signals in *A. thaliana* (Fig. 3d, left) and about 6 and 5 knob-specific signals in *Z. mays* (Fig. 3D, right) were detected by standard and indirect CRISPR-FISH, respectively. In addition, 47 and 45 nuclei out of 50 *A. thaliana* nuclei showed centromere-specific signals and 45 and 43 nuclei out of 50 *Z. mays* nuclei showed knob specific-signals generated by standard and indirect CRISPR-FISH, respectively (Supplementary Fig. 3c). Overall, the quantification results showed a satisfactory labeling efficiency of indirect CRISPR-FISH using a 3'biotin-labeled tracrRNA compared with standard CRISPR-FISH.

**Fig. 4:**
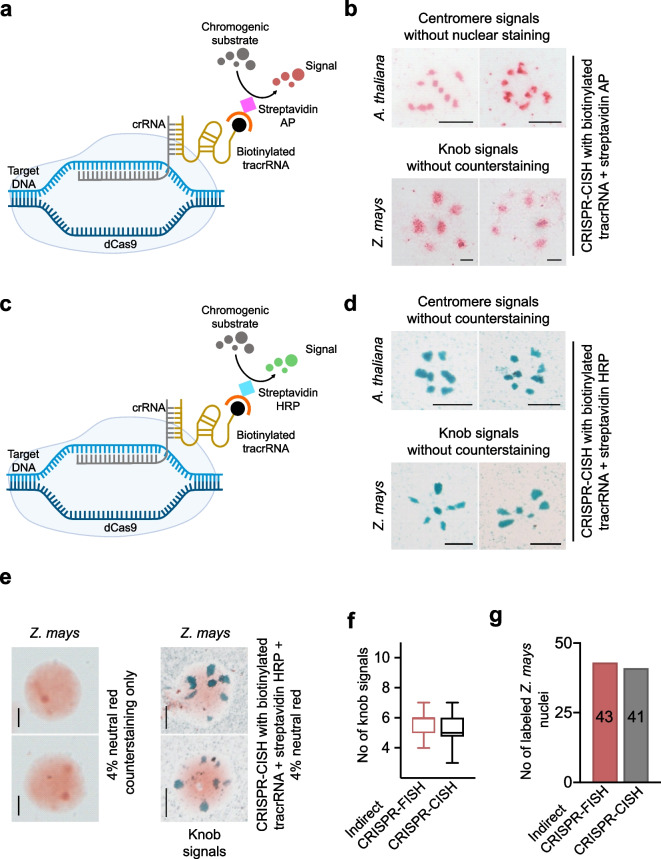
CRISPR-Cas9-mediated chromogenic in situ detection of repeat sequences in fixed samples. **a** Schematic of CRISPR-CISH for labeling repeat sequences using biotinylated tracrRNA and streptavidin alkaline phosphatase (AP) and application of red chromogenic substrate develops red colored precipitate at RNP bound sites. **b** CRISPR-CISH-based non-fluorescent labeling of centromeres (top) and knob repeats (bottom) in *A. thaliana* and *Z. mays* nuclei without counterstaining using streptavidin AP and red chromogenic substrate. Red-colored spots indicate centromere and knob-specific signals. **c** Schematic of CRISPR-CISH for labeling repeat sequences using biotinylated tracrRNA and streptavidin–horseradish peroxidase (HRP) and application of green chromogenic substrate develops green colored precipitate at RNP bound sites. **d** CRISPR-CISH-based non-fluorescent labeling of centromeres (top) and knob repeats (bottom) in *A. thaliana* and *Z. mays* nuclei without counterstaining using streptavidin HRP and green chromogenic substrate. Green-colored spots indicate centromere and knob-specific signals, respectively. (**e**, right) CRISPR-CISH labeling of knob repeats in *Z. mays* nuclei counterstained with 4% neutral red and (**e**, left) counterstained only as a control with no labeling. Scale bars, 5 µm. **f** Comparison of labeling efficiency between two methods, number of knob signals generated per nucleus by indirect CRISPR-FISH and CRISPR-CISH methods, *n* = 50 nuclei. Upper and lower whiskers indicate the maximum and minimum number of signals observed. The black line in the middle of each box plot indicates the median. (**g**) Bar graph comparing the efficiency of indirect CRISPR-FISH and CRISPR-CISH methods. The number of nuclei with labeled knob repeats on fixed 2 C nuclei of *Z. mays* is shown. *n* = 50 nuclei

Finally, we employed the 3'biotin-labeled tracrRNA in combination (I) with streptavidin-conjugated alkaline phosphatase (Fig. 4a) or (II) streptavidin-conjugated horseradish peroxidase (Fig. 4c) and corresponding chromogenic substrates. We termed this strategy “CRISPR-CISH”, a CRISPR-Cas9 mediated chromogenic in situ hybridization method. The application of the streptavidin-conjugated alkaline phosphatase enzyme and the red substrate for signal detection in formaldehyde-fixed *A. thaliana* and *Z. mays* nuclei using centromere and knob-specific gRNA, respectively, resulted in a specific labeling of the repeats (Fig. 4b). Although the signals generated by CRISPR-CISH in combination with alkaline phosphatase were specific, the structure of the signals was a bit fuzzy. In contrast, the application of the peroxidase enzyme along with the green chromogenic substrate resulted in clear and sharper green-colored centromere and knob signals (Fig. 4d). After the chromogenic reaction, *Z. mays* nuclei were counterstained in 4% neutral red for 2 min. The use of a conventional bright-field microscope revealed strong green knob-specific signals on light orange-stained nuclei (Fig. 4e, right). As a control *Z. mays* nuclei were counterstained without CRISPR-CISH (Fig. 4e, left). To assess the efficiency of CRISPR-CISH, we compared the number of knob repeat-specific signals generated by indirect CRISPR-FISH and CRISPR-CISH. About 5 knob-specific signals were counted after indirect CRISPR-FISH and CRISPR-CISH, respectively (Fig. 4f). In addition, 43 and 41 nuclei out of 50 *Z. mays* nuclei showed knob-specific signals generated by indirect CRISPR-FISH and CRISPR-CISH, respectively (Fig. 4g).

**Fig. 5:**
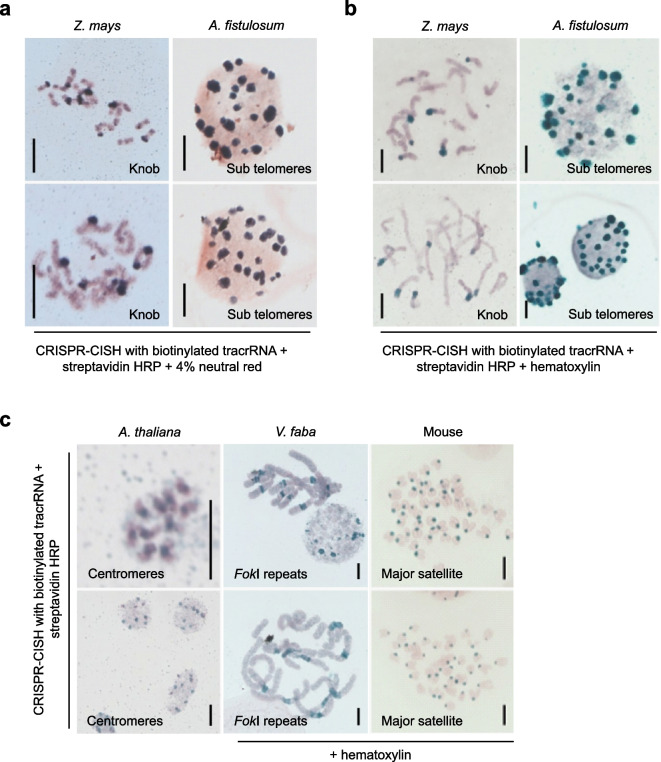
CRISPR-Cas9-mediated chromogenic in situ detection of repetitive sequences in ethanol: acetic acid-fixed chromosomes and nuclei. Non-fluorescent visualization of knob and sub telomere *repeats* on *Z. mays* chromosmes (left) and *A. fistulosum* nuclei (right) using CRISPR-CISH in combination with (**a**) neutral red and (**b**) hematoxylin staining. **c** Visualization of centromeres, *Fok*I and major satellite repeats in *A. thaliana*, *V. faba* and mouse, respectively, on ethanol: acetic acid-fixed chromosomes using CRISPR-CISH. *V. faba* and mouse samples were stained with hematoxylin. Scale bars, 5 μm

Finally, to test whether also CRISPR-CISH labels repeats on conventionally 3:1 (ethanol: acetic acid)-fixed chromosomes and nuclei, we employed *Z. mays* chromosomes and *A. fistulosum* nuclei for labeling the knob and subtelomere repeats, respectively. After CRISPR-CISH application, either 4% neutral red or 100% hematoxylin was used to stain the chromatin. CRISPR-CISH successfully labeled the knob repeats at terminal regions of a subset of chromosome arms in *Z. mays* (Fig. 5a,b, left) and the subtelomeres repeats in *A. fistulosum* nuclei (Fig. 5a,b, right).

The application of 4% neutral red resulted in both species in light red counterstained chromatin and dark-colored CRISPR-CISH signals. In difference, the use of hematoxylin produced green-colored CRISPR-CISH signals and light red counterstained chromatin (Fig. 5a,b). Further, using CRISPR-CISH in combination with hematoxylin also successfully labeled the centromere repeats of *A. thaliana, Fok*l repeats in the mid-arm position of the long arms of all *V. faba* chromosomes and the major satellite repeats on mouse chromosomes (Fig. 5c). As a positive control, streptavidin-conjugated FITC was used to detect indirect CRISPR-FISH specific signals in 3:1-fixed *A. thaliana*, *Z. mays*, *V. faba*, *A. fistulosum*, and mouse chromosomes (Supplementary Fig. 4).

In conclusion, CRISPR-CISH enables the successful labeling of repeats in both formaldehyde or 3:1 fixed nuclei and chromosomes. Conventional bright-field microscopy is sufficient for the analysis of the specimens. The application of horseradish peroxidase-based detection is better than alkaline phosphatase because it results in more distinct and clear chromogenic signals with less background. A detailed protocol for the application of CRISPR-CISH in educational institutes and science outreach settings is provided (Supplementary Fig. 5).

## Discussion

Standard CRISPR-FISH uses a fluorescently labeled tracrRNA to visualize repetitive DNA sequences in fixed samples (Ishii et al. 2019). In our study, we developed a non-fluorescent CRISPR-ISH method and evaluated two indirect labeling strategies: using anti-Cas9 antibodies and biotinylated tracrRNA. The Cas9 antibody approach was less efficient, likely due to formaldehyde fixation, which may alter protein conformations and mask epitopes, hindering the accessibility of the antibody (Bogen et al. 2009; O’Leary et al. 2009). In contrast, the application of biotinylated tracrRNA resulted in a higher labeling efficiency. The strong and specific interaction between biotin and streptavidin remained stable even under stringent fixation and washing conditions, contributing to the enhanced performance of this method (Kolodziej et al. 2009). When using biotinylated tracrRNA, CRISPR-CISH produced much clearer signals with the streptavidin horseradish peroxidase conjugate than with the alkaline phosphatase conjugate. This could be due to the larger size of the alkaline phosphatase (approximately 86 kDa), which may hinder its ability to penetrate the chromatin (Bradshaw et al. 1981). In contrast, horseradish peroxidase, which is only 40 kDa in size, likely penetrates the chromatin more efficiently (Rennke and Venkatachalam 1979).

Compared to other tested chromatin counterstains, haematoxylin and neutral red showed optimal staining, providing better contrast between signals and nuclei or chromosomes. Unfortunately, none of the nonfluorescence dyes stained *A. thaliana* nuclei uniformly, indicating the need for further investigations by screening additional dyes in future studies.

Unlike standard FISH, CRISPR-CISH is a fast and convenient method that offers several advantages over conventional DNA detection techniques. In particular, the application time of CRISPR-CISH is much shorter due to the rapid DNA binding of Cas9 (Ishii et al. 2019). In contrast to standard FISH, which requires an expensive fluorescence microscope, CRISPR-CISH only needs a conventional light microscope to visualize the labeled DNA. In addition, the chromogenic signals generated by CRISPR-CISH are sufficiently strong to be visualized with a 40× lens without fading over time, allowing specimens to be archived for long periods. Moreover, CRISPR-CISH uses mild conditions that make it easy and safe to conduct hands-on investigations in educational institutions or laboratories where expensive fluorescence microscopes and specialists may not be available.

In educational settings, CRISPR-CISH can be a powerful tool to inspire students by enabling them to visualise DNA repeats under a conventional bright-field microscope. To make CRISPR-CISH more accessible in schools, it could be combined with the Foldscope, a paper-based origami microscope that offers 140 × to 350× magnification and is available as a classroom kit (https://foldscope.com) (Cybulski et al. 2014). Additionally, using pre-assembled dCas9/gRNA RNP complexes to label DNA eliminates the need for GMO-related permissions and raises no ethical concerns, making it an ideal tool for classroom applications.

However, the application of CRISPR/Cas9 is limited by the requirement for the NGG PAM sequence at the target site, which is necessary for designing the gRNA. This limitation can be overcome by using engineered Cas9 variants that work with different PAM sequences (reviewed by (Collias and Beisel 2021). To date, CRISPR-CISH has efficiently labeled high-copy genomic DNA repeats, but it is less efficient at labeling low- and single-copy sequences. To improve its utility for low-copy sequences, CRISPR-CISH could potentially be combined with the dCas9-ALFA-tag approach, which has recently demonstrated increased signal intensities (Potlapalli et al. 2024). Despite these limitations, CRISPR-CISH holds significant potential for future research and diagnostic applications in less-funded settings, where it can facilitate the investigation of basic chromosome biology.

## Supplementary Information

Below is the link to the electronic supplementary material.

Supplementary file 1 (DOCX 44 KB)

Supplementary file 2 (PDF 407 KB)

## Data Availability

No datasets were generated or analysed during the current study.
