# Supplementary material for: CRISPR-CISH: an in situ chromogenic DNA repeat detection system for research and life science education
Source: Chromosome Res. 2025 Apr 22;33(1):7. doi: 10.1007/s10577-025-09767-1 (PMC12011966; doi:10.1007/s10577-025-09767-1)
Supplement: Supplementary file 1 — (DOCX 44 KB) [file 10577_2025_9767_MOESM1_ESM.docx]

**Supplementary figures**

**Supplementary Fig. 1:** (a) Non-fluorescent labeling of DNA repeats by combining CRISPR-ISH with immunoassay on formaldehyde fixed nuclei of *V. faba* (upper) and *A. fistulosum* (lower), labeling *Fok*I and sub telomeres, respectively, without counterstaining. Dark red spots indicate *Fok*I and sub-telomeric specific signals, respectively. (b) Control labeling of FokI and sub-telomeric repeats on formaldehyde-fixed nuclei of *V. faba* (upper) and *A. fistulosum* (lower) using CRISPR-FISH. Scale bars, 5 μm.

**Supplementary Fig. 2: Comparison of different nuclear stains on fixed nuclei.** (a) Staining of *Z. mays*, *A. fistulosum* and *V. faba* nuclei with 2% methyl green, 2% methylene green, 4% neutral red, 100% hematoxylin (left) and 100% methylene blue (right) solution for 2 min each. (b) Staining of *A. thaliana* nuclei with 100% hematoxylin (right) and 100% methylene blue (left) solution. Hematoxylin and methylene blue also stained the chromocenters. Scale bars, 5 μm.

**Supplementary Fig. 3: Labeling of DNA repeats using standard CRISPR-FISH and DNA FISH.** Visualization of centromere and telomere repeats on formaldehyde fixed *A. thaliana* and *N. benthamiana* with (a) standard CRISPR-FISH (top) using Atto550 tracrRNA and (b) DNA FISH using biotin-labeled oligo probes and detected with streptavidin FITC, respectively. DNA stained with DAPI (blue). Scale bars, 5 μm. (c) Bar graph comparing the efficiency of standard CRISPR-FISH and indirect CRISPR-FISH methods. The number of nuclei with centromere and knob repeats on fixed 2C nuclei of (left) *A. thaliana* and (right) *Z. mays* is shown. n =50 nuclei.

**Supplementary Fig. 4: Indirect CRISPR-FISH labeling on ethanol:acetic acid fixed nuclei and chromosomes.** Visualization of centromere, knob, *Fok*I, sub telomere and major satellite repeats in ethanol: acetic acid fixed chromosomes of *A. thaliana*, *Z. mays*, *V. faba*, *A. fistulosum* and mouse. Labeling was performed using indirect CRISPR-FISH with biotinylated tracrRNA, followed by detection with streptavidin FITC. DNA stained with DAPI (blue). Scale bars, 5 μm.

**Supplementary Fig. 5:** Schematic illustration of the CRISPR-CISH protocol.

**Supplementary Table**

Supplementary Table: List of crRNA sequences and probe DNA

| **Species** | **crRNA / oligo** | **crRNA sequence** | **Target** | **Source** |
| --- | --- | --- | --- | --- |
| ***A. thaliana*** | Cent | 5`- TTGAGAAGCAAGAAGAAGGT -3` | centromere repeats | (Ishii et al. 2019) |
| ***A. thaliana*** | At.Tel | 5`- GGGTTTAGGGTTTAGGGTTT -3` | telomere repeats | (Ishii et al. 2019) |
| ***Z. mays*** | Knob2 | 5`-AAGGAAACATATGTGGGGTGAGG-3` | 180 bp Knobs repeats | (Němečková et al. 2019) |
| ***A. fistulosum*** | AfiSat-375_1 | 5` -TGACCGCTGTAAGCCGTCAA- 3` | sub telomeric repeats | (Irifune et al. 1995) |
| ***A. cepa*** | AceSat-375_2 | 5` -CTTCGGAGGGCCATAACTCT- 3` | sub telomeric repeats | (Irifune et al. 1995) |
| ***V. faba*** | Vf *Fok1a* | 5` -CGAGATTTTTGTTACTCCAATGG- 3` | Fok repeats | (Fuchs et al. 1994) |
| ***M. musculus*** | MS1a | 5′-CAGTTTTCTCGCCATATTCCAGG-3′ | Major satellite | (Potlapalli et al. 2020) |
| ***A. thaliana*** | Cent oligo | ACACCATGAAAGCTTTGAGAAGCAAGAAGAAGGTTGGTTA | centromere repeats | (Murata et al. 1994) |
| ***A. thaliana*** | Telo  oligo | GGGTTTAGGGTTTAGGGTTTAGGGTTTAGGGTTT | telomere repeats | (Dreissig et al. 2017) |
