# Supplementary material for: CRISPR-CISH: an in situ chromogenic DNA repeat detection system for research and life science education
Source: Chromosome Res. 2025 Apr 22;33(1):7. doi: 10.1007/s10577-025-09767-1 (PMC12011966; doi:10.1007/s10577-025-09767-1)

Supplementary Fig. 1

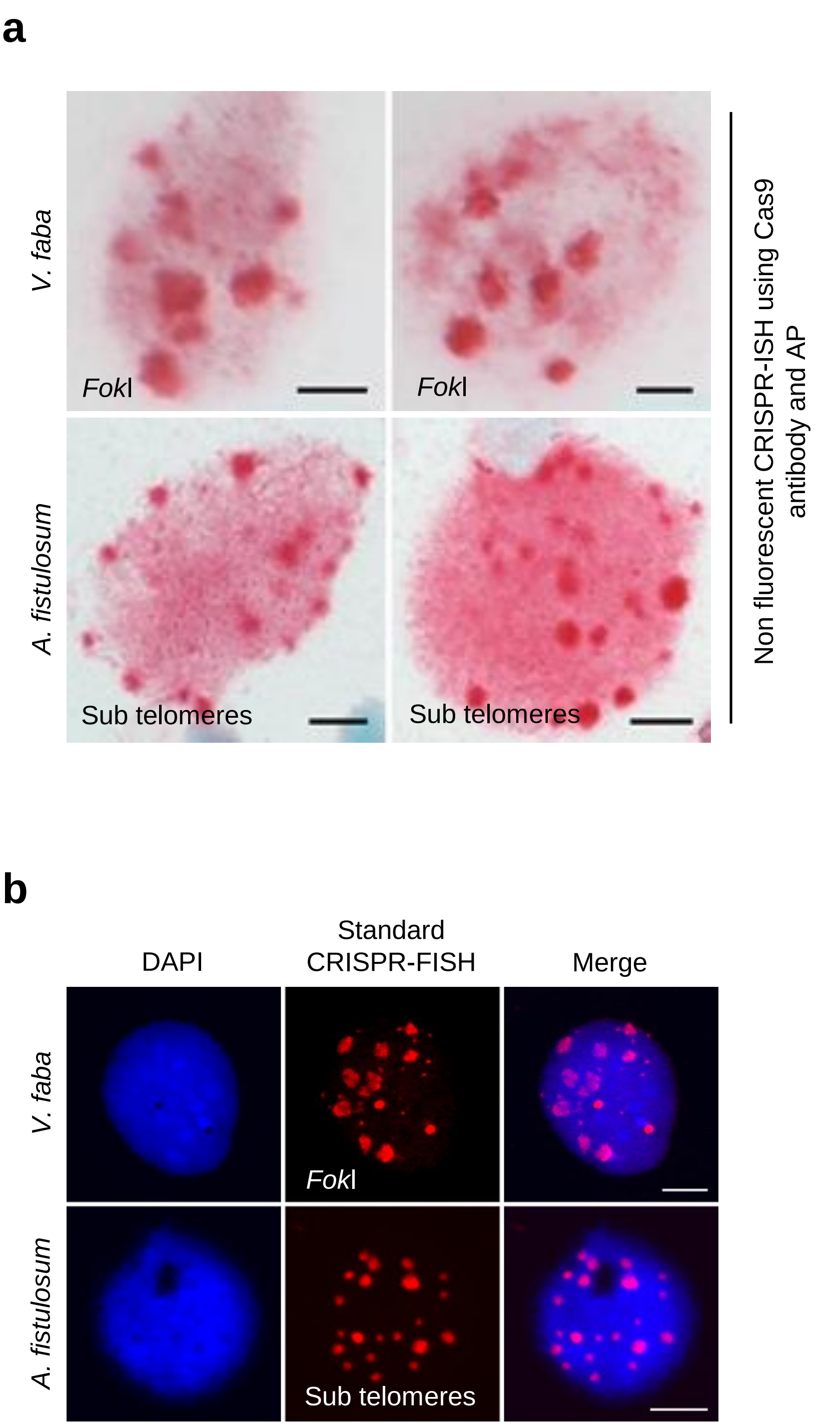

Supplementary Fig. 2

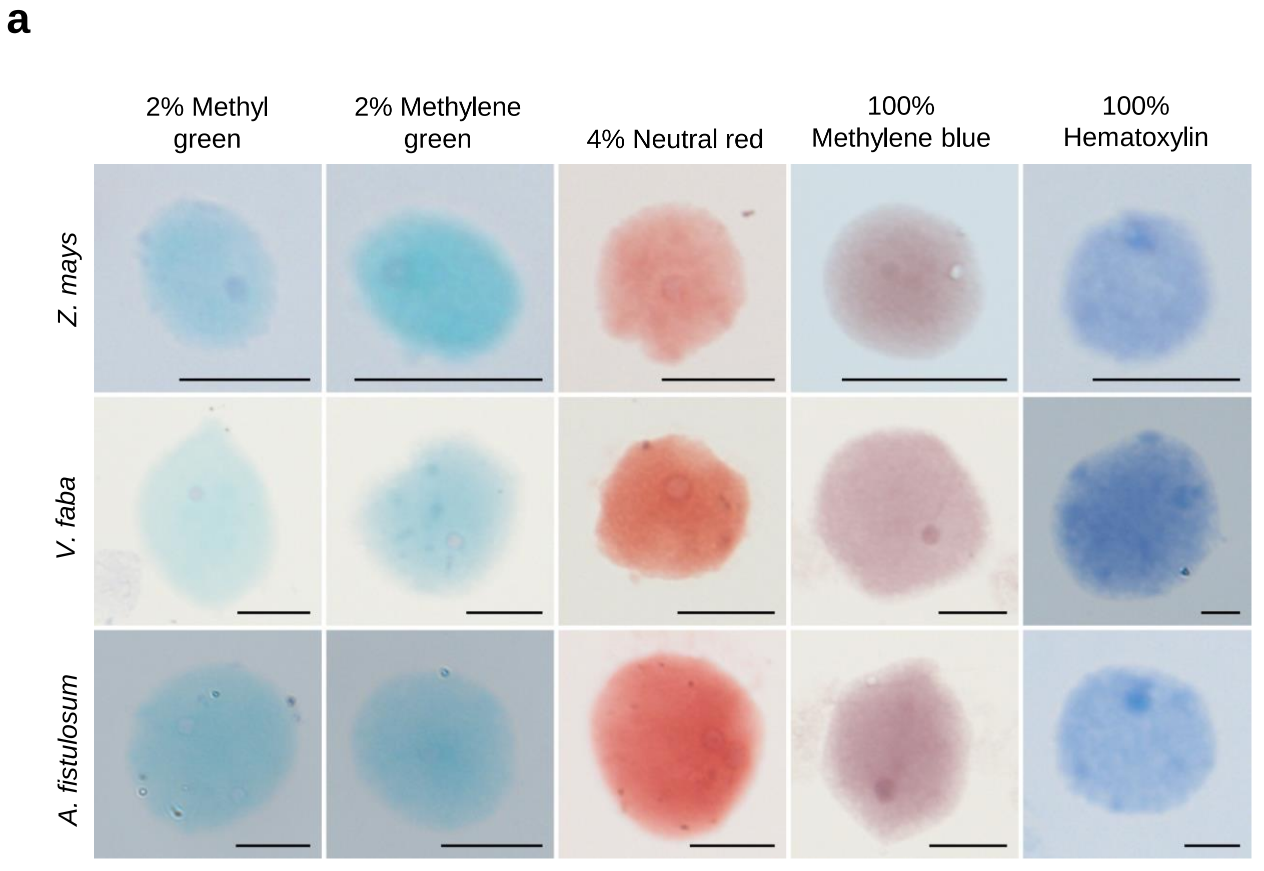

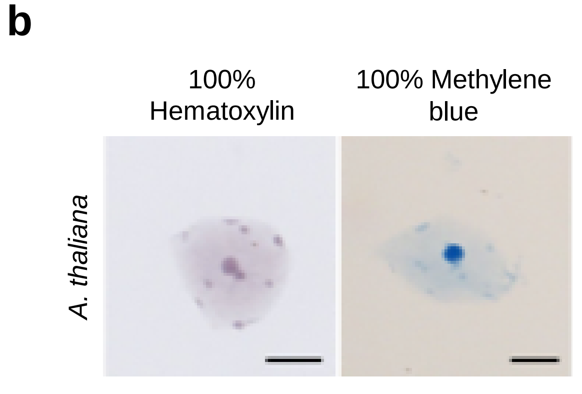

Supplementary Fig. 3

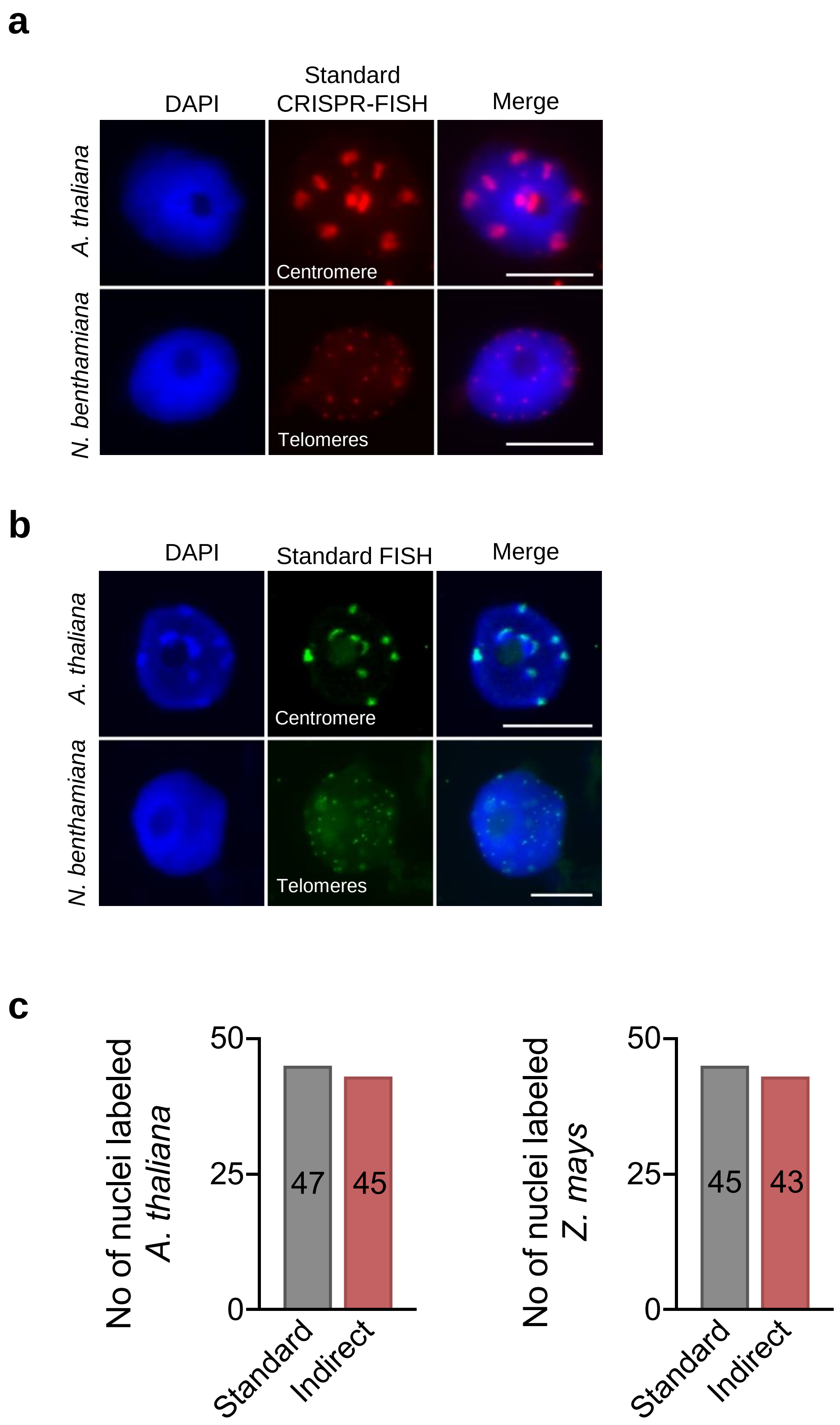

Supplementary Fig. 4

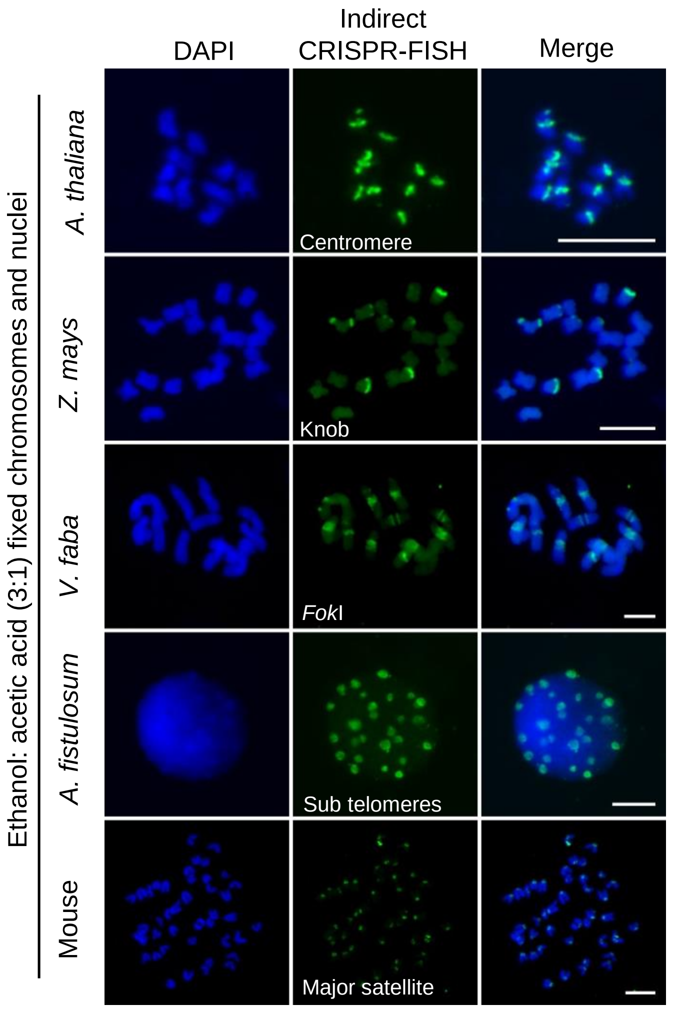

Supplementary Fig. 5

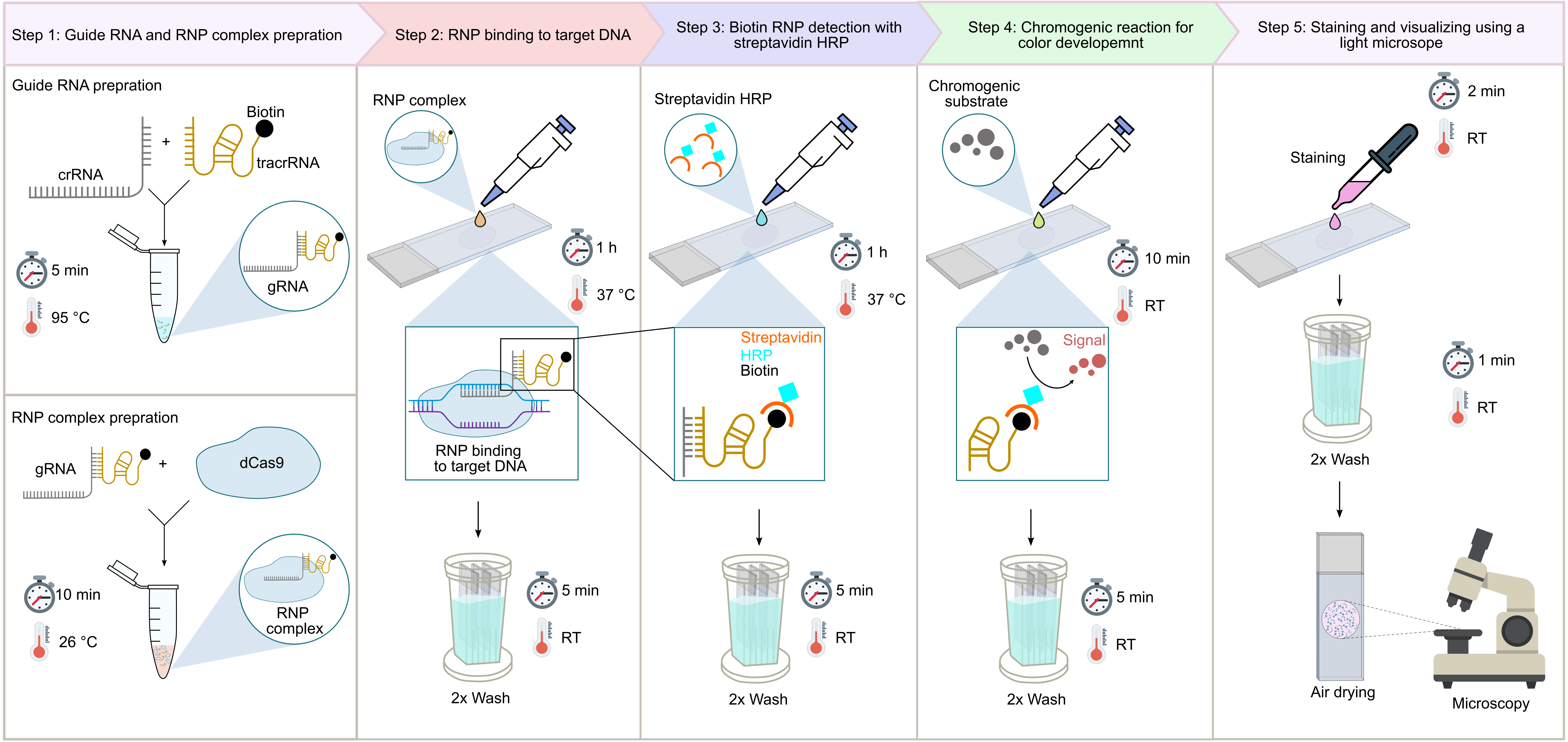

Supplement: Supplementary file 2 — (PDF 407 KB) [file 10577_2025_9767_MOESM2_ESM.pdf]
